# Genetic Analysis of Cachavirus-Related Parvoviruses Detected in Pet Cats: The First Report From China

**DOI:** 10.3389/fvets.2020.580836

**Published:** 2020-11-23

**Authors:** Jun Ji, Wen Hu, Qiang Liu, Kejing Zuo, Guanglin Zhi, Xin Xu, Yunchao Kan, Lunguang Yao, Qingmei Xie

**Affiliations:** ^1^Henan Provincial Engineering Laboratory of Insects Bio-reactor, Henan Provincial Engineering and Technology Center of Health Products for Livestock and Poultry, Nanyang Normal University, Nanyang, China; ^2^Veterinary Laboratory, Guangzhou Zoo, Guangzhou, China; ^3^College of Animal Science, South China Agricultural University, Guangzhou, China

**Keywords:** cachavirus, feline cachavirus, sequence identity, phylogenetic tree, protein mutation, structure prediction

## Abstract

In this study, members of the *Carnivore chaphamaparvovirus species 1*, closely related to a virus previously reported in dog feces named cachavirus was identified for the first time in feces of Chinese cats. Screening tests using rectal swabs from 171 diarrheic and 378 healthy cats collected from Henan, Anhui, and Zhejiang provinces in China revealed two samples from diarrheic cats that were positive for cachavirus, but statistical analysis indicated no association between the presence of the virus and clinical signs (*p* > 0.05). Subsequently, two partial genome sequences [from nucleotides 479–4123, according to the strains from dogs (cachavirus)] of the two strains from cats (cachavirus-cat1 and -cat2) were amplified. The NS1 and VP1 sites of cachavirus-cat1 and -cat2 shared a high identity of 91.9 and 97.0% with reported cachaviruses, respectively, but lower identity of 74.8 and 73.2% with another carnivore chaphamaparvovirus named fechaviruses detected in cats, respectively, indicated the two strains might origin from dogs. These findings improve our understanding of the diversity and tropism of viruses in *Carnivore chaphamaparvovirus species 1* which now include both dogs and now cats viruses.

## Introduction

Parvoviruses belong to the family *Parvoviridae*, which are small non-enveloped viruses with single-stranded DNA (ssDNA) genomes that are ~4–6 kilobases (kb) in length (1). The novel subfamily *Hamaparvovirinae* comprises the current genera *Hepandensoviruses, Ichthamaparvovirus, Penstyldensovirus*, and *Brevidensovirus*. Vertebrate infecting *Chaphamaparvovirus* genus is also included in that subfamily according to the latest taxonomic criteria of the International Committee on Taxonomy of Viruses.

In recent years, chaphamaparvoviruses (ChPV) have been detected in several hosts. Bat (*Eidolon helvum*) parvovirus 2 (EHPV2) was first reported in bats in 2013 (2), and then it was identified in other bats such as the fruit bat (*E. helvum*) parvovirus 1 (3) and bat (*Desmodus rotundus*) parvovirus (4). Studies have reported that expanding its host spectrum of chaphamaparvoviruses, these viruses were also identified in mammals such as mice (5, 6), simian (7), swine (8), and Tasmanian devils (9). Other chaphamaparvoviruses were also identified in birds, including red-crowned cranes (10), turkeys (11), peafowl (12), and chickens (13). More notably, two strains of Cachavirus-1A (MH893826) and Cachavirus-1B (MK448316), were also identified in dogs in the United States in 2019, and the cachavirus which belonged to *Carnivore chaphamaparvovirus species 1* had three open reading frames (ORFs) encoding a 663-amino acid non-structural protein 1 (NS1), a 504-amino acid capsid protein (VP1) and a 210-amino acid putative nucleoprotein (NP) (14). More recently, another ChPV named fechavirus was detected in feline outbreak of diarrhea and vomiting and reported in May 2020 (15). To better understand the host range of members of the *Carnivore chaphamaparvovirus species 1*, we collected samples from healthy and diarrheic cats to investigate whether the cachavirus or fechavirus was present in cats in China.

## Materials and Methods

### Sample Collection and DNA/RNA Extraction

Rectal swabs of 171 diarrheic and 378 healthy cats were collected from pet hospitals in Henan, Hubei, Anhui, and Zhejiang provinces, China, during 2018–2019. The collected swabs were washed with 1 mL phosphate-buffered saline for nucleic acid extraction. According to the manufacturers' instructions, the virus DNAs/RNAs were extracted using the EasyPure Viral DNA/RNA Kit (TransGen Biotechnology, Inc., Beijing, China) and stored at −80°C until use.

### Virus Screening and Cultivation

The extracted DNAs/RNAs were examined by polymerase chain reaction (PCR) or Reverse Transcription-Polymerase Chain Reaction (RT-PCR) for Feline bufavirus (FBuV), Feline coronavirus (FcoV), FPV, fechavirus, and cachavirus, as reported previously (14–18). Primer sequences for screening tests are listed in Supplementary Table 1. Supernatants from the diluted fechavirus or cachavirus-positive samples were filtered through a 0.22-μm membrane and inoculated onto a monolayer of *Crandell Rees* Feline Kidney (CRFK, originating from cats) cells and *Madin–Darby* Canine Kidney (MDCK, originating from dogs) cells at 37°C in 5% CO_2_. Viral growth was evaluated through five serial passages of CRFK and MDCK cells by monitoring the onset of cellular cytopathic effects. Culture lysates and supernatants were also collected for the pathogen screening described above.

### Cloning and Partial Genome Sequencing

The partial genomes of the cachavirus positive samples were amplified. The primers were designed with reference to Cachavirus-1A as shown in Supplementary Table 2. Gene amplification was performed by PCR in a 20-μL reaction mixture comprising a template DNA (>100 ng/μL), 6 pmol of upstream/downstream primers, PrimerSTAR HS DNA polymerase, and a supporting reaction buffer (TaKaRa Biotechnology Co., Ltd., Dalian, China). Sequence amplification was performed under the following cycling conditions. Initial denaturation at 95°C for 3 min, followed by 34 cycles of denaturation at 95°C for 30 s, annealing at 55°C for 30 s, and extension at 72°C for 1 min, with a final extension at 72°C for 10 min. The obtained amplicons were then cloned into a pMD18-T easy vector (TaKaRa Biotechnology Co., Ltd., Dalian, China) for future sequencing (Syn-Biotechnology, Suzhou, China). PCR and genome sequencing were performed at least three times.

### Identity and Phylogenetic Analysis

The partial genome sequences of the cachaviruses in the two cats were submitted to the GenBank database under the accession No. MN928790 - MN928791. NS1 and VP1 of the strains of this study were compared with ChPVs detected in the canine and other animals using the ClustalW method in MEGA 7.0 to analyze their identity. The phylogenetic tree was constructed for NS1 and VP1 genes to analyze the evolution relationships between the obtained ChPVs with the reference strains using the MEGA 7.0 software and the maximum likelihood method with the pairwise deletion option and 1,000 bootstrap replicates.

### Protein Mutation and Structure Prediction of NS1 and VP1

IBS (Illustrator for Biological Sequences) was used to draw the general relationship of cachavirus from cats compared with Cachavirus-1A. Referring to the Cachavirus-1A and Cachavirus-1B strains, the obtained sequences were aligned for amino acid site mutations. According to the different amino acid sites, the mutated amino acid sequences were modeled in the SWISS-MODEL (https://swissmodel.expasy.org/interactive), and postmodeling, Pdb files were constructed with the PyMOL software for collation and preservation.

### Statistical Analysis

Fisher's exact test was used to compare the frequency of cachavirus between healthy and diarrheic cats. Statistical analyses were performed using GraphPad Prism 8.0 (San Diego, CA, USA). A *p* < 0.05 was considered statistically significant.

## Results

### Sample Positive Rate and Coinfection Situation

Following the screening tests, 2/171 diarrheic cats and 0/378 healthy cats were PCR positive for cachavirus DNA, and statistical analysis measured a *p*-value of 0.097, suggested no association between the presence of the virus and clinical signs (*p* > 0.05). The co-infection of FPV was also identified. The details of the clinical information of the cachaviruses (cat1-CNC181031 and cat2-CNC190520) are presented in Supplementary Table 3.

### Viral Cultivation

Until the fifth generation, CPE was only observed in the cultured cells inoculated by CNC190520 with FPV coinfection and the cells were only positive for FPV, but the DNA of cachavirus and other pathogen were not detected in the culture lysates or supernatant by PCR.

### Identity Analysis of NS1 and VP1 Genes of the Two Cachavirus Strains From Cats

The identity of feline cachaviruses with Cachavirus-1A and Cachavirus-1B was 91.9% for NS1 and 97.0% for VP1. However, the identity of the two cachavirus strains from cats was only 74.6–74.8% for NS1 and 72.8–73.2% for VP1 of the Feline chaphamaparvovirus, respectively. The identity of two cachaviruses was 24.0–73.5% for NS1 and 22.0–66.7% for VP1 compared with the other reference ChPV strains. The NS1 and VP1 of cachaviruses detected in cats shared the highest identity with Bat parvovirus isolate (MG693107), which was 73.5% for NS1 and 66.7% for VP1. The detailed data is shown in Table 1.

**Table 1:** Sequence identities of the study strains of cachavirus with the members of the CHPV.

**Virus strains**	**Accession no**.	**Sequence identity (%)**		**Genetic distance of VP1**
		**NS1(aa)**	**VP1(aa)**	
*Desmodus rotundus*parvovirus	NC032097	59.2	57.3–57.4	0.557–0.565
*Eidolon helvum*parvovirus	JX885610	61.0–61.2	NA	NA
Turkey parvovirus	KF925531	51.0–51.2	36.2–36.5	0.713–0.718
Simian parvo-like virus	KT961660	24.0	22.0–22.4	0.770–0.780
Porcine parvovirus 7	KU563733	28.6–28.7	27.9–28.1	0.596–0.602
Rat parvovirus 2	KX272741	57.0–57.2	56.1–56.3	0.585
Parvoviridae sp.	KY312548	52.6–53.0	48.5	0.481–0.494
Murine chappavovirus	MF175078	29.5–29.7	36.9–37.0	0.592–0.600
Bat parvovirus	MG693107	73.5	66.7	0.676–0.696
Chicken chappavovirus 1	MG846441	50.1–50.2	NA	NA
Chicken chappavovirus 2	MG846442	47.8–48.1	52.0–52.3	0.488–0500
Tasmania devil-associated chappavovirus 1	MK513528	42.6–42.8	49.0–49.1	0.497–0.513
Cachavirus-1A	MH893826	91.8–91.9	96.8–97.0	0–0.010
Cachavirus-1B	MK448316	91.9	96.7–97.0	0–0.010
Unclassified chaphamaparvovirus	MN794869	74.6–74.8	72.8–73.1	0.032–0.034
Feline chaphamaparvovirus	MN396757	74.7–74.8	72.8–73.2	0.032–0.034

*NA, not available*.

### Phylogenetic Tree Analysis for NS1 and VP1 Genes

**Figure 1:**
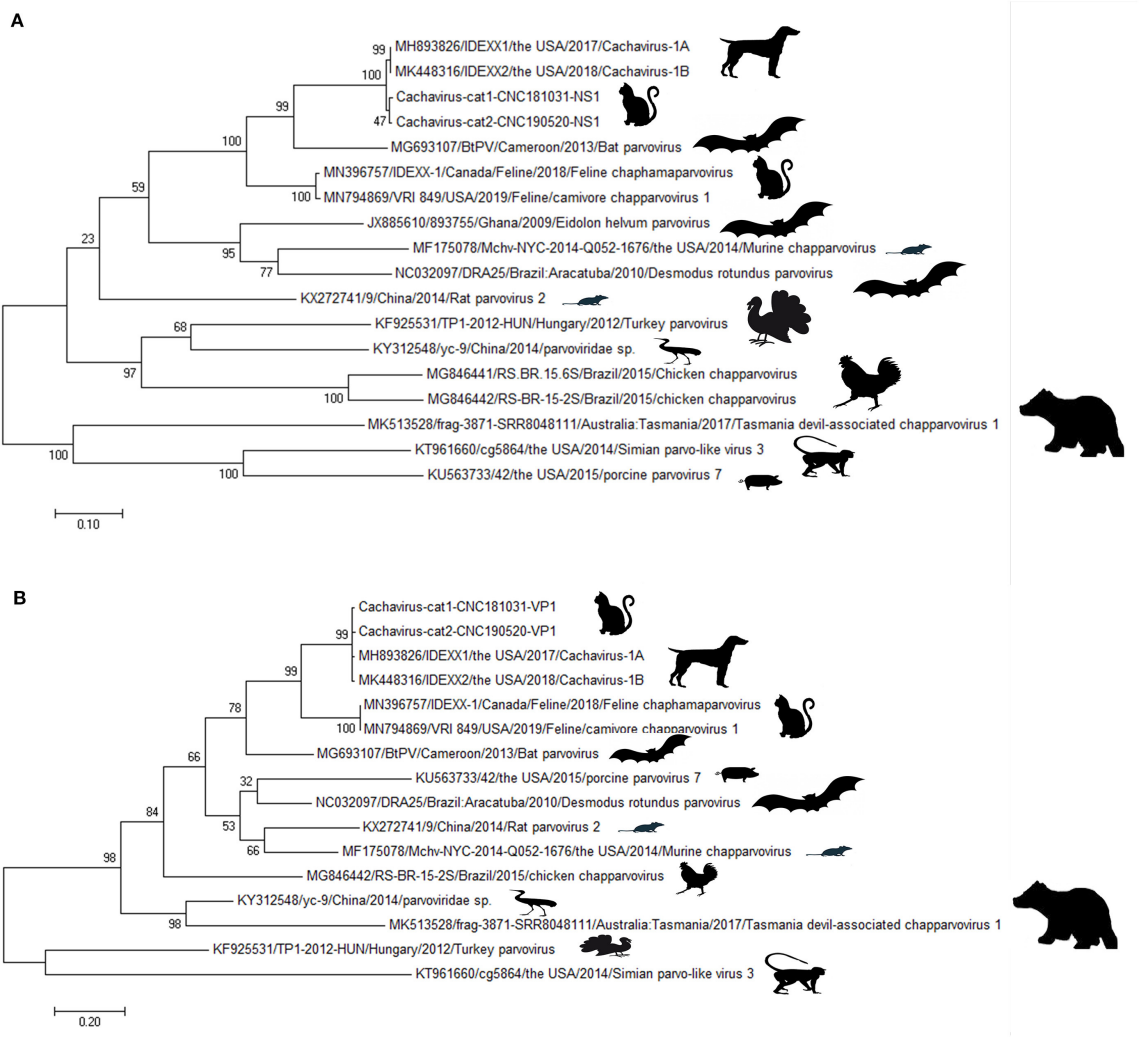
Phylogenetic tree of the NS1 and VP1 genes in ChPV. **(A)** Phylogenetic tree of the NS1 gene in cachavirus from cats; **(B)** phylogenetic tree of the VP1 gene in cachavirus from cats.

Phylogenetic trees were constructed for the NS1 (Figure 1A) and VP1 (Figure 1B) of ChPVs. As displayed in the phylogenetic trees based on *NS1* and *VP1*, the reference strains and the two cachavirus strains from cats were generally divided into two major branches. The two cachaviruses from cats were distant from fechaviruses, but more closely related to the reference strains Cachavirus-1A and Cachavirus-1B.

### Mutation Analysis and Structure Prediction of NS1 and VP1 Genes

Through comparing the complete genome construction of Cachavirus-1A in the NCBI database and feline cachavirus (partial genome sequences, 479–4123) (Supplementary Figure 1), suggested the genomic structure of feline cachavirus was consistent with that of Cachavirus-1A, with two major ORFs encoding *NS1* and *VP1*; the putative nucleoprotein (*NP*) was 210-amino acid long. The cachaviruses detected in cats shared 99.1–99.2% *NP* identity with Cachavirus-1A. The ATP-binding Walker loop motif GPSNTGKS was also present in *NS1* ORF of the two strains similar to that of Cachavirus-1A.

**Figure 2:**
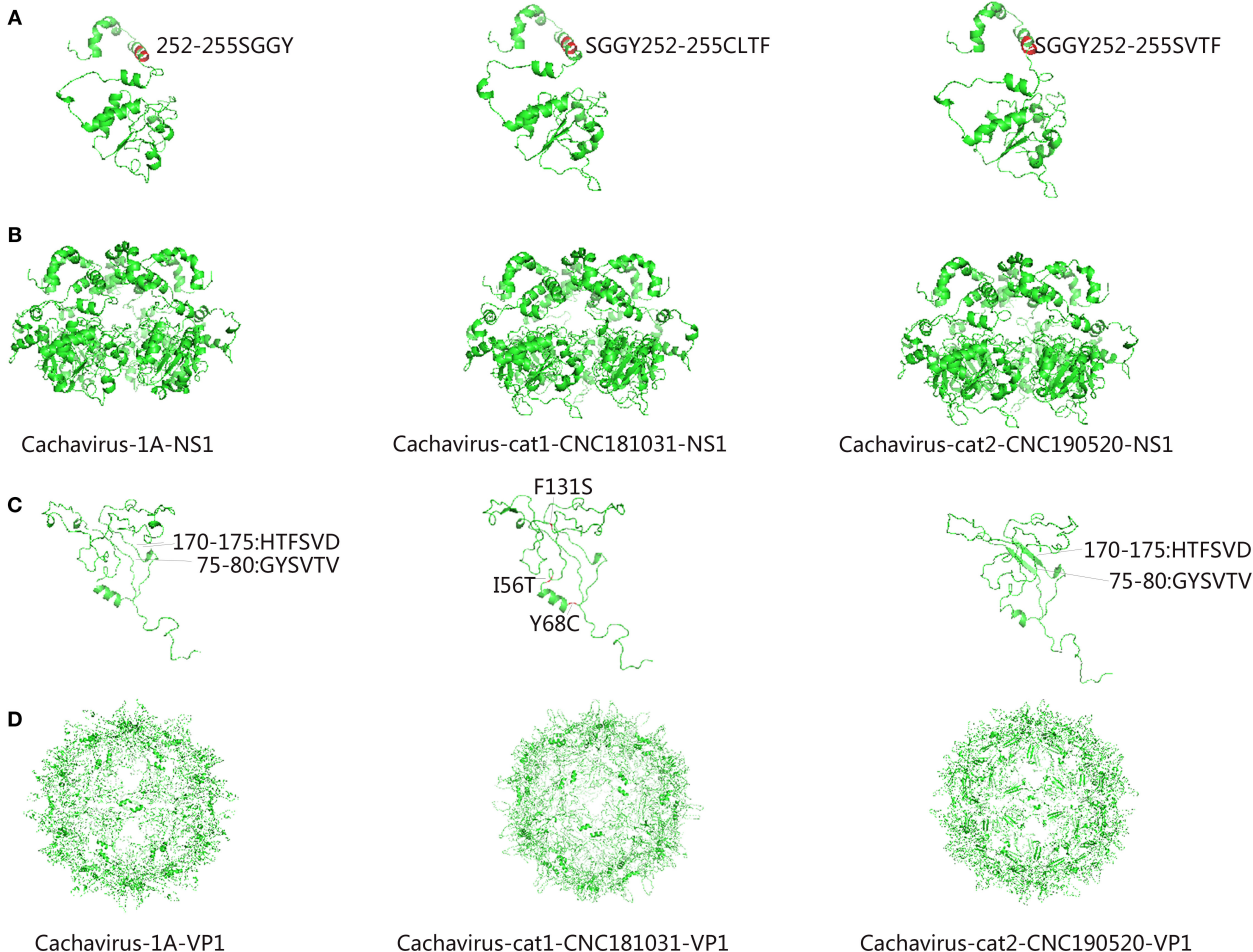
Predicted tertiary structural model of cachavirus in NS1 and VP1. **(A,B)** Tertiary structural model of NS1; **(C,D)** Tertiary structural model of VP1.

Comparing the amino acid sites of the NS1 and VP1 of feline cachavirus and cachavirus, nine main mutant sites were identified for NS1: Ser252Cys(1/2), Gly253Val(1/2), Gly253Leu(1/2), Gly254Thr(2/2), Tyr255Phe(2/2), Thr477Ile(1/2), Arg544Gln(1/2), Gly603Arg(2/2), and Arg607Gly(2/2) (Table 2A), and 10 mutant sites for VP1: Tyr22His(1/2), Ile56Thr(1/2), Tyr68Cys(1/2), Phe131Ser(1/2), Val265Ile(2/2), Gln365Arg(1/2), Asp402Asn(1/2), Arg449Lys(2/2), His484Pro(1/2), and Val493Ile(1/2) (Table 2B). The tertiary structures of the NS1 and VP1 of the two strains were also predicted, and the obtained tertiary structures were compared with Cachavirus-1A. Predicted structure differences caused by the mutant sites are displayed in Figure 2.

**Table 2A:** Statistics of the main amino acid mutation sites in the NS1 capsid protein of cachavirus in Chinese strains (“CN”; this study) and reference strains.

**Strains**	**Substitution of amino acid residues in NS1**							
	**252**	**253**	**254**	**255**	**477**	**544**	**603**	**607**
Cachavirus-1A	S	G	G	Y	T	R	G	R
Cachavirus-1B	S	G	G	Y	T	R	G	G
Cachavirus-cat1-CNC181031	C	L	T	F	T	Q	R	G
Cachavirus-cat2-CNC190520	S	V	T	F	I	R	R	G

**Table 2B:** Statistics of the main amino acid mutation sites in the VP1 capsid protein of cachavirus in Chinese strains (“CN”; this study) and reference strains.

**Strains**	**Substitution of amino acid residues in VP1**									
	**22**	**56**	**68**	**131**	**265**	**365**	**402**	**449**	**484**	**493**
Cachavirus-1A	Y	I	Y	F	V	Q	D	R	H	V
Cachavirus-1B	Y	I	Y	F	I	Q	D	R	H	V
Cachavirus-cat1-CNC181031	Y	T	C	S	I	Q	N	K	H	V
Cachavirus-cat2-CNC190520	H	I	Y	F	I	R	D	K	P	I

## Discussion

In this study, we detected cachavirus infections in Chinese pet cats. Among healthy and diarrheic cats collected from Henan, Hubei, and Zhejiang provinces, China, only two samples from diarrheic cats were positive for cachavirus. The infection rate was ~1.17% (2/171) in diarrheic cats, suggesting that the virus had not been widespread in cats. In addition, cachavirus was found to co-infect with FPV in one sample. Statistical analysis indicated no association between the presence of the virus and diarrheic signs (*p* > 0.05). To date, report about pathogenicity of the ChPVs in vertebrate hosts is still very limited. Mouse kidney parvovirus (MKPV) has been demonstrated to cause a kidney disease known as inclusion body nephropathy among laboratory mice populations (19). Meanwhile, high prevalence of murine ChPV in murine liver tissue, suggesting it is a gastrointestinal agent (6). Despite the lack of disease association the only two infected cats were also both sick. It is therefore not excluded that cachavirus may be associated with a small fraction of diarrhea in cats. Herein, larger investigation and animal inoculation experiments are needed to determine whether cachavirus may be pathogenic.

In terms of the phylogenetic trees based on NS1 and VP1, the cachaviruses from dogs and described here from cats, belong to the same branch, which suggested the relationship as well as genetic distances between these ChPVs. However, based on the identity and phylogenetic analysis, these two strains tested in the Chinese cats were quite different from the other feline chapparvovirus named fechavirus (15), but more closely related to the virus previously found in dogs in the United States. These results were in consistent with the genetic distance analysis, indicated that the cahaviruses in cats and dogs may have a recent common origin although what host species had it first cannot be determined. Unfortunately, we only amplified the partial genome sequence comprising 479–4123 of the predicted genome, although we attempted to design several primer sets to accomplish the amplification of the 5′-UTR of cachavirus from cats based on the sequence of Cachavirus-1A and Cachavirus-1B. It was suspected that these sequences of cachavirus from cats that were not amplified were quite different from cachavirus detected in the United States (14). The specific reason requires further successful isolation methods and second-generation sequencing to obtain the complete sequence and confirm the difference.

Compared with Cachavirus-1A and Cachavirus-1B, the cachavirus from cats demonstrated changes in amino acid sites, and some mutations changed the tertiary structure modeling of the two major viral proteins as predicted. From the structure prediction diagram of NS1 in Figure 2, the two obtained cachaviruses demonstrated minimal differences in tertiary structure modeling with Cachavirus-1A, and some mutation sites of the two strains did not change the NS1 protein structure of the virus. For the mutation of the VP1 structural protein, this possibly indicates that four mutations of Cachavirus-cat1-CNC181031-VP1 are the cause of these changes (Ile56Thr, Tyr68Cys, Phe131Ser, and Asp402Asn). The tertiary structure modeling of Cachavirus-cat1-CNC181031-VP1 was significantly changed, indicating that mutations at these amino acid sites may cause changes in the structure of the protein. However, further studies are warranted to investigate whether site mutations would lead to changes in its function and pathogenesis. Simultaneously, the structure of Cachavirus-cat2-CNC190520-VP1 also changed compared with Cachavirus-1A, suggesting that the changes at Tyr22His and Gln365Arg lead to the changes in its structure. Similarly, further evidence is required to ascertain whether these mutations really cause these structural changes.

In conclusion, we believe that this study identified a novel parvovirus—cachavirus—in Chinese pet cats for the first time. The findings of this study enhance our understanding of the tropism of different members of the *Carnivore Chaphamaparvovirus 1 species* which now appears to infect both dogs and cats.

## Data Availability Statement

The datasets presented in this study can be found in online repositories. The names of the repository/repositories and accession number(s) can be found in the article/Supplementary Material.

## Ethics Statement

Sample collection protocols were approved by the cat's owner and Animal Welfare and Ethics Committee of Nanyang Normal University (No. 14027).

## Author Contributions

JJ and XX designed the study. WH and QL performed the sampling collection and wrote the manuscript. KZ and GZ performed the clinical investigations and necropsies. YK and LY performed the molecular genetic studies. QX helped to draft the manuscript. All authors have read and approved the final manuscript. All authors contributed to the article and approved the submitted version.

## Conflict of Interest

The authors declare that the research was conducted in the absence of any commercial or financial relationships that could be construed as a potential conflict of interest.

## Supplementary Material

Supplementary Figure 1 Genome construction of Cachavirus-1A and cachavirus from cats.
