# Supplementary figures and images for: Genetic Analysis of Cachavirus-Related Parvoviruses Detected in Pet Cats: The First Report From China
Source: Front Vet Sci. 2020 Nov 23;7:580836. doi: 10.3389/fvets.2020.580836 (PMC7719813; doi:10.3389/fvets.2020.580836)

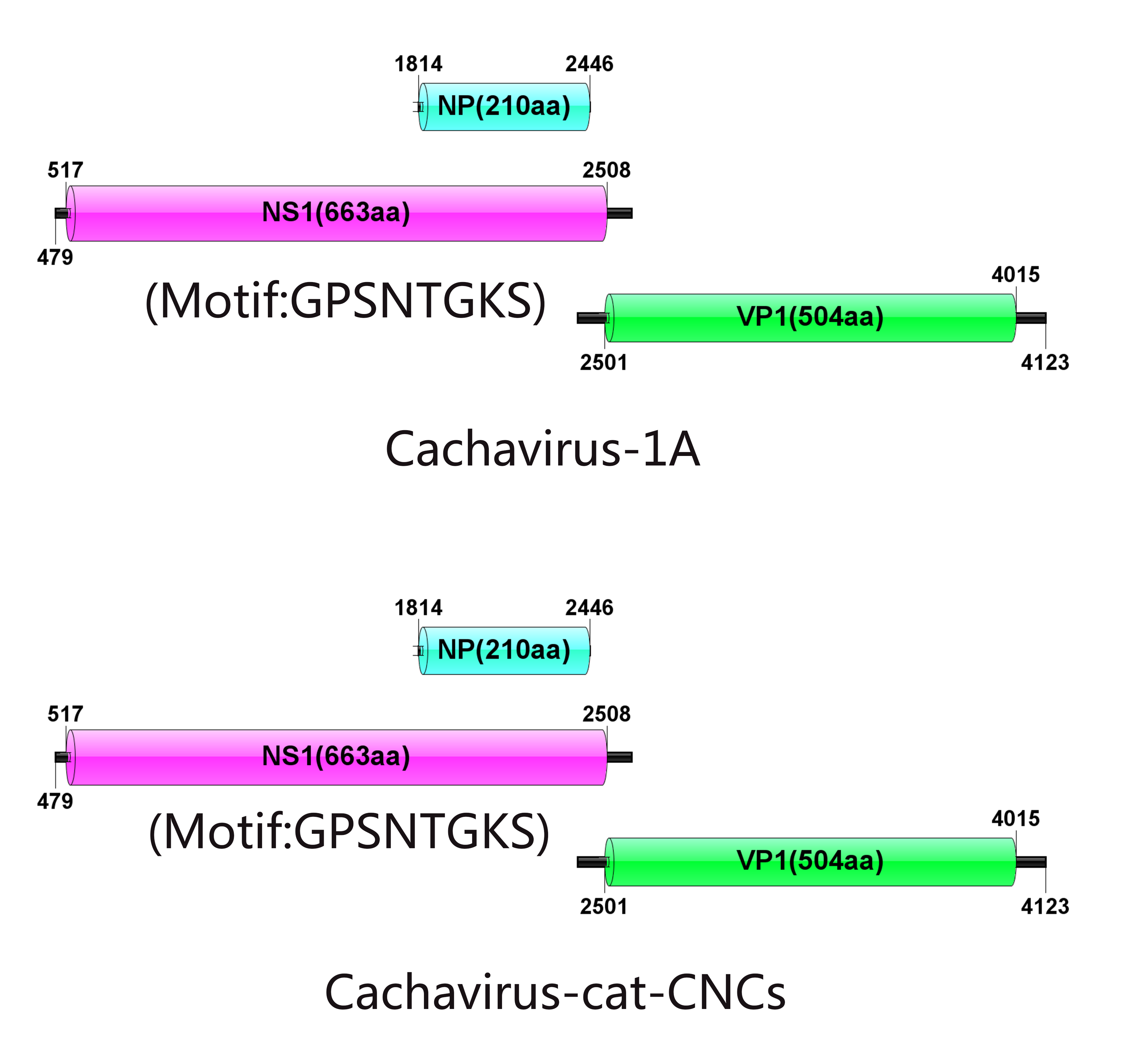

Supplement: Supplementary Figure 1 — Genome construction of Cachavirus-1A and cachavirus from cats. [file Image_1.TIF]
