# Supplementary material for: Genetic Analysis of Cachavirus-Related Parvoviruses Detected in Pet Cats: The First Report From China
Source: Front Vet Sci. 2020 Nov 23;7:580836. doi: 10.3389/fvets.2020.580836 (PMC7719813; doi:10.3389/fvets.2020.580836)
Supplement: Supplementary file 2 [file Data_Sheet_1.doc]

Table 1: Primers for detecting FBuV, FcoV, FPV, fechavirus and cachavirus

| **Primer** | **Sequence (5′-3′)** | **Target (bp)** | **Reference** |
| --- | --- | --- | --- |
| *FBuV-F* | CTGGTTTAATCCAGCAGACT | 202 | (16) |
| *FBuV-R* | TGAAGACCAAGGTAGTAGGT |
| *FcoV-F1* | GGCAACCCGATGTTTAAAACTGG | 205 | (17) |
| *FcoV-R1* | GCTCTTCCATTGTTGGCTCGTC |
| *FcoV-F2* | CCGAGGAATTACTGGTCATCGCG | 191 |
| *FcoV-R2* | CACTAGATCCAGACGTTAGCTC |
| *FPV-F* | GCACATCAAGATACAGGAAG | 800 | (18) |
| *FPV-R* | CCTTAACATATTCTAAGGGCAA |
| *Cachavirus-F1* | CAACTAGCCGAATGCAGGGA | 323 | (14) |
| *Cachavirus-R1* | CGATAACATCCCCGGACTGG |
| *Cachavirus-F2* | AGCTCAGTTTGGCCCAGATC | 224 |
| *Cachavirus-R2* | AGAGGGATCGCTGGATCTGT |  |
| *FechaF1* | GGTGCGACGACGGAAGATAT | 332 | (15) |
| *FechaR1* | CAACACCACCATCTCCTGCT |
| *FechaF2* | GCTGCAGTTCAGGTAGCTCA | 310 |
| *FechaR1* | CAACACCACCATCTCCTGCT |

**Table S2.**  Primers for partial cachavirus genome amplification

| Primers | Sequences (5′-3′) | Target (bp) | Information |
| --- | --- | --- | --- |
| CxChPV-1F | GTTACAGCAACCGTTGACTCGT | 1371 | 479–500^†^  1829–1850 |
| CxChPV-1R | CTACAGCATTCGCAGCTCTCGT |
| CxChPV-2F | GCTATTATGATTTAGGAGAACGCTT | 1098 | 1055–1079  2133–2153 |
| CxChPV-2R | CTGGTTCGTATCCCGTCGCTA |
| CxChPV-3F | CTCCTGCACCTCAGTTAGCG | 935 | 2118–2137  3036–3053 |
| CxChPV-3R | GCCATACAGCCGATCCAC |
| CxChPV-4F | TGCACAAGATGATCTATACGAA | 1294 | 2827–2848  4100–4123 |
| CxChPV-4R | GGATACACAGGCGCCAGTACAGTA |

^†^Genome position according to Cachavirus-1A

**Table S3.** Details of the two cachavirus sequences identified in this study

| Sample | Healthy status | Age | Region | Date | Coinfection |
| --- | --- | --- | --- | --- | --- |
| Cachavirus-cat1-CNC181031 | Diarrhea | 2 m | Henan | October 31, 2018 | / |
| Cachavirus-cat2-CNC190520 | Diarrhea | 4 m | Henan | May 20, 2019 | FPV |

FPV: feline panleukopenia virus
